# Ecologic Niche Modeling and Potential Reservoirs for Chagas Disease, Mexico.

**DOI:** 10.3201/eid0807.010454

**Published:** 2002-07

**Authors:** A. Townsend Peterson, Victor Sánchez-Cordero, C. Ben Beard, Janine M. Ramsey

**Affiliations:** * Natural History Museum, Lawrence, Kansas, USA; † Universidad Nacional Autónoma de México, D.F., México; ‡ Centers for Disease Control and Prevention, Atlanta, Georgia, USA; § and Centro de Investigaciones sobre Enfermedades Infecciosas (CISEI), Cuernavaca, Morelos, México

**Keywords:** disease reservoirs, methods, Chagas disease, Triatoma, Neotoma

## Abstract

Ecologic niche modeling may improve our understanding of epidemiologically relevant vector and parasite-reservoir distributions. We used this tool to identify host relationships of *Triatoma* species implicated in transmission of Chagas disease. Associations have been documented between the protracta complex (*Triatoma*: Triatominae: Reduviidae) with packrat species (*Neotoma* spp.), providing an excellent case study for the broader challenge of developing hypotheses of association. Species pairs that were identified coincided exactly with those in previous studies, suggesting that local interactions between *Triatoma* and *Neotoma* species and subspecies have implications at a geographic level. Nothing is known about sylvatic associates of *T. barberi*, which are considered the primary Chagas vector in Mexico; its geographic distribution coincided closely with that of *N. mexicana*, suggesting interaction. The presence of the species was confirmed in two regions where it had been predicted but not previously collected. This approach may help in identifying Chagas disease risk areas, planning vector-control strategies, and exploring parasite-reservoir associations for other emerging diseases.

Chagas disease is caused by the parasitic protozoan *Trypanosoma cruzi* and transmitted by blood-feeding insects in the family *Reduviidae*, subfamily Triatominae. Chagas disease is an important cause of illness and death throughout the Americas, affecting 16–18 million persons. While an estimated 100 million persons in 21 countries in the New World live in endemic areas and are at risk for infection, the disease is principally a zoonotic infection, in which sylvatic mammals serve as reservoir hosts and zoophilic triatomine species as vectors.

The protracta species group consists of seven species (Triatoma protracta, T. peninsularis, T. sinaloensis, T. neotomae, T. barberi, T. nitida, and T. incrassata); T. protracta contains five subspecies: T. p. protracta, T. p. woodi, T. p. navajoensis, T. p. zacatecensis, and T. p. nahuatlae (1,2). This group is restricted to the southwestern United States and Mexico. Previous studies have demonstrated high host specificity in this species group, involving woodrats or packrats (Neotoma spp.) (1). Whereas host associations of Triatoma are often complex, the protracta group shows remarkable host specificity and geographic distributions suggestive of host-ectoparasite cospeciation.

A new tool in the study of geographic phenomena in ecology and systematics is ecologic niche modeling of primary occurrence data (data placing a particular species in a particular state) (3). In general, the approach involves a machine-learning algorithm for discovering associations between point-occurrence data and sets of electronic maps summarizing environmental/ecologic dimensions that may or may not be important in limiting species’ geographic distributions. These associations constitute an approximation of species’ fundamental ecologic niches (the conjunction of ecologic conditions in which a species is able to maintain populations without immigration) (4) and hence provide a basis for understanding numerous ecologic and geographic phenomena related to species distributions.

We applied ecologic niche modeling to identify host relationships of *Triatoma* species and subspecies implicated in the transmission of Chagas disease. Previous studies by Ryckman (1) provide an ideal test case: hypotheses of association developed based on the modeling approach can be tested independently by using associations identified in Ryckman’s detailed field studies. If successful, this approach would be invaluable in identifying host relationships for species for which detailed information is not available, for stratifying Chagas disease risk areas, and for planning the operational aspects of vector control strategies.

## Methods

### Point-Occurrence Information

Distribution data for members of the protracta species group were obtained from multiple sources (5–11; state vector control programs in Morelos and San Luis Potosí, unpub. data). Distribution data for *Neotoma* woodrats occurring in mainland Mexico (excluding offshore islands) were drawn from the Atlas of the Mammals of Mexico (Comisión Nacional para el Conocimiento y Uso de la Biodiversidad, unpub. data) and Hall (12) (textual localities only, georeferenced by hand from 1:50,000-scale maps to approximately 1 km precision). All occurrence data were georeferenced to the nearest 0.001° and organized in Excel 2000 (Microsoft Corp., Redman, WA) spreadsheets for analysis.

### Distribution Modeling

Ecologic niches and potential geographic distributions were modeled with the Genetic Algorithm for Rule-Set Prediction (GARP) (13–15), a complex computer application that provides a broader, more objective approach than traditional Geographic Information System (GIS)-based approaches (3) but which yields GIS coverages as output. In general, the procedure focuses on modeling ecologic niches (16). Specifically, GARP relates ecologic characteristics of known occurrence points to those of points randomly sampled from the rest of the study region and develops a series of decision rules that summarizes those factors associated with the species’ presence (3).

All modeling in this study was carried out on a desktop implementation of GARP in a beta-testing stage (R. Scachetti-Pereira, unpub. data). In this software package, occurrence points are divided evenly into training and test data sets. The GARP program works in an iterative process of rule selection, evaluation, testing, and incorporation or rejection: a method is chosen at random from a set of possibilities (e.g., logistic regression, bioclimatic rules), applied to the training data, and a rule is developed (“evolved,” in the terminology of genetic algorithms). At each iteration in the program’s processing, predictive accuracy is then evaluated based on 1,250 points resampled from the test data and 1,250 points randomly sampled from the study region as a whole. Rules may evolve in several ways that genetic algorithms use to mimic DNA evolution (e.g., point mutations, deletions, crossing over). The program uses the change in predictive accuracy from one iteration to the next to evaluate whether a particular rule should be incorporated into the model; the algorithm runs 1,000 iterations or until convergence.

The desktop GARP implementation offers much-improved flexibility (17) in choice of predictive environmental/ecologic GIS data coverage. In this case, we used 11 data layers summarizing elevation; slope; aspect (from the U.S. Geological Survey’s HYDRO 1K data set, http://edcdaac.usgs.gov/gtopo30/hydro/); and aspects of climate, including cloud cover; daily temperature range; mean annual precipitation; maximum, minimum, and mean annual temperatures; vapor pressure; and wind speed (annual means 1960–1990; from the Intergovernmental Panel on Climate Change, http://www.ipcc.ch/). Analysis was limited to Mexico, because of the availability of distribution data for *Triatoma*, and cell resolution was set at 1x1 km. GARP’s predictive abilities have been tested and proven under diverse circumstances (3,17–23).

To optimize model performance, we developed 100 replicate models of each species’ ecologic niche based on random 50-50 splits of available occurrence points. Unlike previous applications, which either used single models to predict species’ distributions (20) or summed multiple models to incorporate model-to-model variation (19), we used a new procedure (Peterson et al., unpub. data) for choosing best subsets of models. The procedure is based on the observations that 1) models vary in quality, 2) variation in models involves an inverse relationship between errors of omission (leaving out true distribution area) and commission (including areas not actually inhabited), and (3) best models (as judged by experts blinded to error statistics) are clustered in a region of minimum omission of independent test points and moderate area predicted (an axis related directly to commission error). The relative position of the cloud of points relative to the two error axes provides an assessment of the relative accuracy of each model. To choose best subsets of models, we eliminated all models that had nonzero omission error based on independent test points, calculated the average area predicted present in these zero-omission points, and identified models that were within 1% of the overall average. For species or subspecies for which fewer than 10 distribution points were available (which would have a weak extrinsic test of model quality) we developed 20 replicate models based on all points available and summed them as a “best” distribution hypothesis. Five species or subspecies were omitted because of the small sample size (e.g., *T. incrassata*, with one locality known in Mexico) or distribution outside Mexico (e.g., *T. protracta navajoensis*), leaving six taxa for analysis: *T. p. protracta*, *T. p. woodi*, *T. p. zacatecensis*, *T. peninsularis*, *T. sinaloensis*, and *T. barberi*.

Projection of the rule-sets for these models onto maps of North America provided distribution predictions. Model quality was tested by the independent sets of points (50%) set aside before GARP modeling: a chi-square test was used to compare observed success in predicting the distribution of test points with that expected under a random model (proportion of area predicted present x number of test points = expected predictive success if points and predictions were random with respect to each other).

### Host-Ectoparasite Relationships

The protracta species complex is unusual in that Ryckman studied and documented the ectoparasite-host relationships (1), which provided an independent source of information regarding *Triatoma*-rodent interactions. In ArcView (version 3.2, ESRI, Redlands, CA), we calculated areas shared between each species or subspecies of the protracta species complex and each species of *Neotoma* woodrat in Mexico, as well as total modeled distribution areas for each species. We calculated the percent of distribution area (at the highest predicted level in the summed GARP outputs) that each *Triatoma* species or subspecies shares with each *Neotoma* species and assumed the most complete overlap values as suggesting an interaction between the species. We then tested these predictions by using the independent information provided by Ryckman (1), asking if species pairs with highest overlap values coincided with interactions identified in Ryckman’s detailed field studies.

## Results

**Figure 1:**
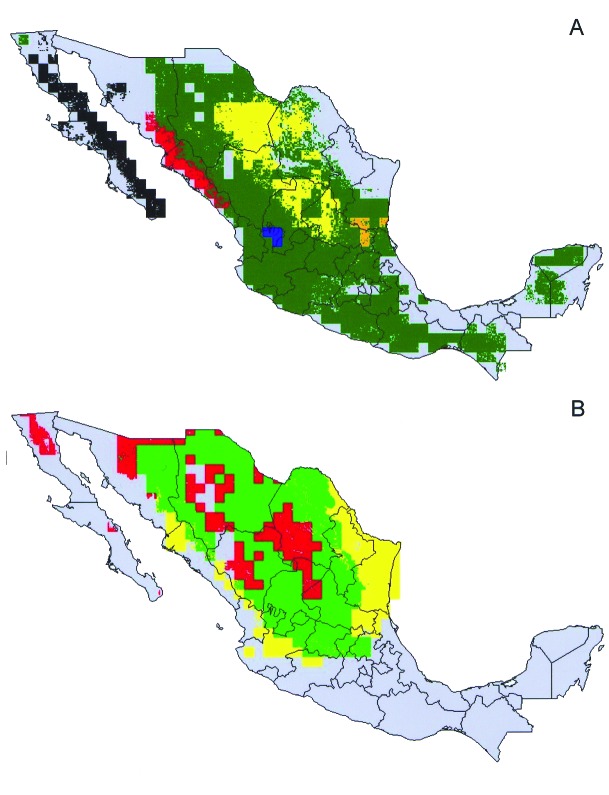
Modeled geographic distributions of *Neotoma* woodrats in mainland Mexico. (A) black = *Neotoma fuscipes*, red = *N. phenax*, green = *N. mexicana* (note that this distribution includes those of other, less widely distributed, species), yellow = *N. goldmani*, blue = *N. palatina*, orange = *N. angustapalata*. (B) red = *N. albigula*, yellow = *N. micropus*, green = *N. albigula* and *N. micropus*.

**Figure 2:**
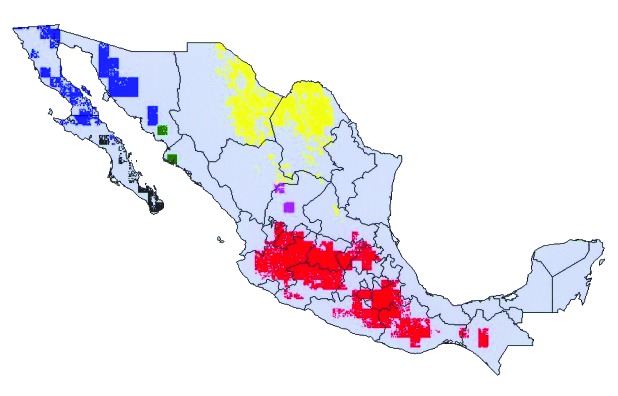
Modeled geographic distributions of members of the protracta species complex: red = *Triatoma barberi*, yellow = *T. p. woodi*, green = *T. sinaloensis*, blue = *T. p. protracta*, black = *T. peninsularis*, and pink = *T. p. zacatecensis*. Only areas predicted for each species at the highest level of confidence (all best-subsets models agree) are shown.

Predictions of distributions for all species and subspecies for which >10 points were available were highly statistically significant (all p<0.001) and indeed coincided well with our understanding of known distributions of both insects (JMR and CBB) and woodrats (VSC). For example, *T. barberi* was predicted to extend broadly across central and southern Mexico, and populations were predicted in several regions (e.g., northern and eastern Michoacán and southern Hidalgo) for which previous occurrence data were not available. After analysis, this species was collected at Tlamaya, Hidalgo (J.C. Noguez-García, pers. comm.), and in northeastern Michoacán (E. Navarro, pers. comm.). Based on the random 50% resampling, in which half the data are set aside to provide an independent test of model quality, the 12 best-subset GARP models for this species were highly statistically significant (average p<10^-114^). The overprediction of the distribution area for this species in Chiapas represents prediction into areas not inhabited for historical reasons, such as speciation, extinction, or limited dispersal ability (18). Tests for *Neotoma* species (Figure 1) were also highly significant (*N. albigula* p<10^-18^, *N. goldmani* p<10^-16^, *N. lepida* p<10^-11^, *N. mexicana* p<10^-5^, *N. micropus* p<10^-13^, and *N. phenax* p<10^-73^); some areas of overprediction (e.g., *N. mexicana* in the Yucatan Peninsula) represent either prediction into areas not inhabited for historic reasons (18) or true overprediction error. Although sufficient point-occurrence data were not available for all species (Figure 2) to permit parallel tests, we are confident in the predictability of our distribution models.

**Figure 3:**
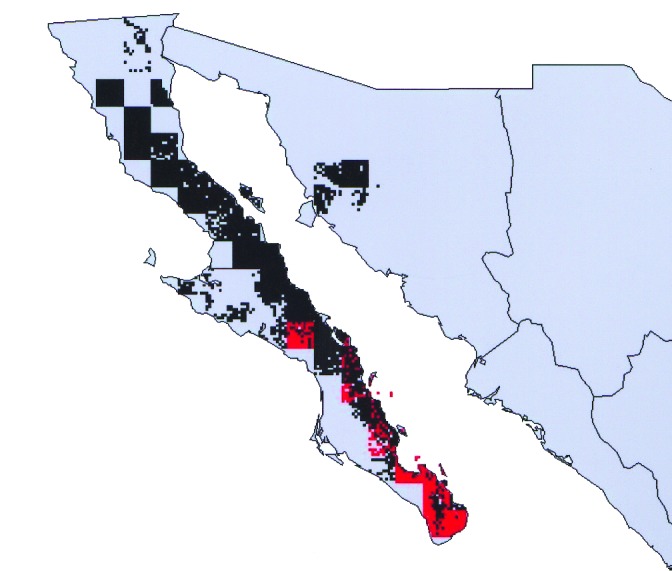
Modeled geographic distributions of *Triatoma peninsularis* (red) and *Neotoma lepida* (black), showing the tight geographic correspondence between the distribution of insect and host mammal. Almost all (93.8%) of the distribution area of *T. peninsularis* overlaps the distribution area of *N. lepida*.

**Figure 4:**
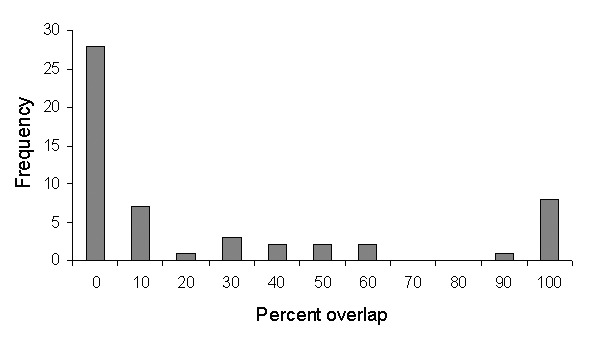
Frequency distribution of areas of overlap between *Triatoma* and *Neotoma* species, expressed as percent of total modeled area (at highest predictive level) of the *Triatoma*.

Overlap of areas in *Triatoma* and *Neotoma* species and subspecies varied considerably (Table). For example, of the total modeled distribution area of *T. peninsularis*, 93.8% of the highest confidence prediction coincided with the distribution of *N. lepida* (Figure 3). Indeed, the overlap values were highly bimodal (Figure 4), suggesting that species’ distributions either coincide or do not overlap, rather than the intermediate peak that might be expected if species did not interact.

**Table:** Distribution of the six species of the protracta complex and nine species of *Neotoma* found in mainland Mexico^a^

	Distribution % of triatomine overlapping *Neotoma*									
	*N. phenax*	*N. palatina*	*N. micropus*	*N. mexicana*	*N. lepida*	*N. goldmani*	*N. fuscipes*	*N. angustapalata*	*N. albigula*	n
*Triatoma barberi*	0.0	2.5	45.1	98.4	0.0	0.1	0.0	0.0	30.0	86
*T. sinaloensis*	**95.5**	0.0	50.8	51.5	0.0	0.0	0.0	0.0	**0.0**	9
*T. peninsularis*	0.0	0.0	0.0	9.0	**93.8**	0.0	82.0	0.0	1.7	9
*T. p. woodi*	0.0	0.0	**94.6**	50.0	0.0	33.3	0.0	0.0	**96.1**	7
*T. p. protracta*	4.4	0.0	6.9	9.2	**24.4**	0.0	**39.3**	0.0	**14.1**	13
*T. p. zacatecensis*	0.0	0.0	100.0	100.0	0.0	24.3	0.0	0.0	**100.0**	9
Points available	31	5	69	103	16	13	4	5	156	

^a^Shown in bold are *Neotoma* species independently identified as hosts for particular *Triatoma* species (1). Distributions are to the highest confidence interval.

The species pairs exhibiting maximum overlap values between *Triatoma* and *Neotoma* species (Table) coincided closely with species associations identified by Ryckman (1). Of the six *Triatoma* analyzed, the maximum overlap values coincided exactly with Ryckman’s identified associations for four of the species. Of the other two species, *T. sinaloensis* shared 95.5 % of its modeled geographic distribution with *N. phenax*, an interaction confirmed by Ryckman. However, our model showed no overlap with *N. albigula*; yet Ryckman found that these two species interacted. Here, the complication is that sample sizes for *T. sinaloensis* were so small (n = 4) that its distribution was underpredicted. Relaxing the criterion for overlap to include areas predicted present by any of the best-subsets models showed an overlap of 33.2% with *N. albigula*. The final *Triatoma* species analyzed, *T. barberi*, was found by Ryckman (1) exclusively around human domiciles; nevertheless, its geographic distribution coincided closely with that of *N. mexicana* (98.4% overlap).

## Discussion

Ecologic niche modeling and distribution prediction with GARP provides a powerful new tool for applications to disease vectors and reservoirs. Even in systems more poorly understood than that examined here, patterns of overlap in geographic or ecologic space can provide initial hypotheses of host associations and disease reservoir or vector species. In this case, we were able to develop rigorous distribution hypotheses for 15 species of mammals and insects that interact in the potential transmission of Chagas disease in Mexico. These distribution hypotheses can form the basis for many applications in this field, including simple distribution prediction (for example, 3,17,20–22), analysis of specific parameters of species’ ecologic niches (20), prediction of species’ distributions across scenarios of climate change (24,25), prediction of species’ invasions (19), assessment of patterns of evolutionary change in ecologic parameters (18; Martinez-Meyer et al., unpub. data), and spatial/epidemiologic stratification of disease endemic areas.

In general, the species pairs identified by ecologic niche modeling and evaluation of overlap of predicted geographic distributions coincided exactly with the interacting species pairs identified in detailed field studies by Ryckman (1). This result suggests that interactions between *Triatoma* and *Neotoma* have implications at a geographic level. That is, a *Triatoma* species does not simply infest the nests of whichever *Neotoma* are present at a particular site; rather, geographic distributions of *Triatoma* species tend to conform closely to those of their *Neotoma* hosts, suggesting a longer term evolutionary relationship. More detailed ecologic and behavioral studies focused on *Triatoma*-*Neotoma* interactions would be invaluable in clarifying the basis for this geographic-scale distribution coincidence.

The only exception to tight coincidence between modeled distribution overlap and Ryckman’s identifications of species interactions was that of *T. sinaloensis* with *N. albigula*. This failure is clearly related to the minimal sample size on which the model for *T. sinaloensis* was based. With increased sample sizes, clearer identification of this interaction should be feasible. Other cases, such as the high overlap of *T. p. zacatecensis* with *Neotoma mexicana* and *N. micropus* (besides the high overlap with its host *N. albigula*), merit close examination in on-site field studies.

**Figure 5:**
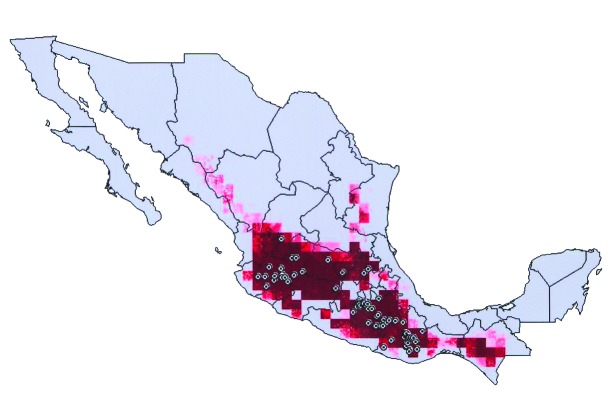
Modeled geographic distribution of *Triatoma barberi*, shown with known occurrence points used to create and test the ecologic niche model. Dark red = 100% of best-subsets models predict presence, medium red = 75% of best-subsets models predict presence, light red = 50% of best-subsets models predict presence, lightest red = any best-subsets model predicts presence.

The most interesting case is that of *T. barberi*. Ryckman (1) identified this species as solely associated with human domiciles and did not find it associated with any rodent species. In Oaxaca, this species has been collected in sylvatic habitats near rock outcroppings, but specific host species have not been identified (7). The species’ extensive geographic distribution (Figure 5) coincided closely (98.4% overlap) with that of *N. mexicana*, at a level that for other *Triatoma* species would almost certainly indicate an interaction. We suggest two possibilities, which are not mutually exclusive: 1) that *T. barberi* does indeed parasitize *N. mexicana* nests, but that additional sampling is necessary to detect this association, or 2) that *T. barberi* may originally have been an ectoparasite of *N. mexicana*, but from that host it made the transition to a domestic or peridomestic existence.

Species’ distribution predictions and our capacity to link these distributions with disease transmission are novel epidemiologic tools. Understanding sylvatic transmission cycles and invasion of peridomestic habitats is an immediate application for niche analysis and modeling. This general approach has potential applications much broader than the protracta species complex and *Neotoma* associations as they relate to Chagas disease transmission. We anticipate addressing host relationships in other *Triatoma* such as the phyllosoma group, the most important complex of vector species of Chagas disease in Mexico and for which host relationships are all but unknown (7). Sylvatic reservoir species for many tropical diseases remain poorly documented (e.g., leishmaniasis, hantavirus pulmonary syndrome, West Nile encephalitis, leptospirosis) and our approach provides a strategy for narrowing the field of possible species and ecologic scenarios.
